# Supplementary material for: Polyketide synthase gene clusters in the ericoid mycorrhizal fungus Oidiodendron maius: genome-wide prediction, expression profiling, and functional characterization of knockout mutants
Source: Front Plant Sci. 2026 Sep 16;17:1924430. doi: 10.3389/fpls.2026.1924430 (PMC13623903; doi:10.3389/fpls.2026.1924430)
Supplement: Supplementary file 1 [file SupplementaryFile1.zip › Supplementary Materials/Supplementary Folder 1.zip!/Oidma1_AssemblyScaffolds_Repeatmasked/knownclusterblast/cluster51/jgi.p_Oidma1_137854_mibig_hits.html]

| MIBiG Protein | Description | MIBiG Cluster | MiBiG Product | % ID | % Coverage | BLAST Score | E-value |
| --- | --- | --- | --- | --- | --- | --- | --- |
| CBF70385.1 | metallo-beta-lactamase\_domain\_protein\_(AFU\_orthologue;\_AFUA\_7G00120) | BGC0000684 | Asperthecin\_biosynthetic\_gene\_cluster | 63 | 97.476340694 | 415 | 4.6e-116 |
| ADI24932.1 | VrtG | BGC0000168 | Viridicatumtoxin\_biosynthetic\_gene\_cluster | 62 | 96.5299684543 | 411 | 6.7e-115 |
| AEN83888.1 | AdaB | BGC0000156 | TAN-1612\_biosynthetic\_gene\_cluster | 62 | 97.1608832808 | 408 | 5.6e-114 |
| EGD99351.1 | metallo-beta-lactamase | BGC0001144 | Neosartoricin\_biosynthetic\_gene\_cluster | 58 | 99.0536277603 | 394 | 1.1e-109 |
| AGO59041.1 | PtaB | BGC0000121 | Pestheic\_acid\_biosynthetic\_gene\_cluster | 51 | 92.7444794953 | 304 | 1.1e-82 |
| EAL89338.1 | metallo-beta-lactamase\_domain\_protein,\_putative | BGC0001403 | Trypacidin\_biosynthetic\_gene\_cluster | 48 | 99.6845425868 | 301 | 1.3e-81 |
| CBF79145.1 | conserved\_hypothetical\_protein | BGC0000013 | Alternariol\_biosynthetic\_gene\_cluster | 50 | 99.3690851735 | 297 | 1.4e-80 |
| ADM34149.1 | metallo-beta-lactamase\_domain\_protein | BGC0001084 | Notoamide\_/\_stephacidin\_biosynthetic\_gene\_cluster | 48 | 94.0063091483 | 292 | 5.9e-79 |
| EAL84396.1 | metallo-beta-lactamase\_domain\_protein | BGC0001118 | Endocrocin\_biosynthetic\_gene\_cluster | 46 | 93.3753943218 | 282 | 4.7e-76 |
| CBF90099.1 | metallo-beta-lactamase\_domain\_protein,\_putative\_(AFU\_orthologue;\_AFUA\_4G14570) | BGC0001271 | Emericellin\_biosynthetic\_gene\_cluster | 50 | 92.7444794953 | 277 | 2.6e-74 |
